# Supplementary material for: Monitoring cognitive resilience in military personnel in extreme operational environments: The role of smart technologies and nutritional strategies—A scoping review protocol
Source: PLoS One. 2025 Jul 10;20(7):e0327649. doi: 10.1371/journal.pone.0327649 (PMC12244529; doi:10.1371/journal.pone.0327649)
Supplement: S1 Appendix — (DOCX) [file pone.0327649.s001.docx]

### **Appendix I: Search strategy**

**Database Used.** A preliminary search was conducted exclusively in PubMed/MEDLINE (via NCBI). The purpose of this initial search was to refine the relevant search terms and evaluate the availability of studies related to military nutrition, cognitive resilience, and smart technologies in military contexts.

**Search Strategy Development.** The search was conducted in two stages:

1. Initial exploratory search: A preliminary search of PubMed was performed to identify appropriate terminology, both MeSH terms and keywords, to ensure a comprehensive search strategy.
2. Refined database search: The search strategy was adjusted based on the results of the initial query to improve retrieval of relevant studies.

**PubMed Search Strategy and Results:**

**Table A1. Summary of search strategies and results**

| 1. | **Initial Integrated Search (4 concepts – 0 hits)** | **Results** |
| --- | --- | --- |
|  | *Search strategy note:*  *The initial 4-concept query retrieved no references. We therefore adopted a revised 3-concept integrated search (row 2) and two complementary thematic searches (row 3). Results from all queries were pooled, deduplicated and screened together.*  ("Military Nutrition"[MeSH] OR "Combat Rations" OR "Dietary Supplements" OR "Operational Rations") AND ("Cognitive Resilience" OR "Cognitive Performance"[MeSH] OR "Mental Resilience" OR "Decision-Making") AND ("Military Personnel"[MeSH] OR "Armed Forces" OR "Soldiers" OR "Military Operations") AND ("Wearable Sensors" OR "Smart Devices" OR "Biomonitoring"). | This search did not retrieve any relevant studies. |
| 2. | **Revised Integrated Search (3 concepts – used for screening)** | |
|  | ("military nutrition" OR "combat rations" OR "dietary supplements") AND ("cognitive resilience" OR "cognitive performance" OR "decision-making") AND ("soldiers" OR "military personnel" OR "armed forces") | 16 Studies identified |
| **3.** | **Thematic Sub-Searches (to maximise sensitivity)** |  |
| 3.1. | **Search on Cognitive Resilience in Military Context** | |
|  | ("Cognitive Resilience" OR "Cognitive Performance" OR "Mental Resilience") AND ("Military Personnel" OR "Armed Forces" OR "Soldiers") | 94 Studies identified |
| 3.2. | **Search on Smart Technologies in Military Context** | |
|  | ("Smart Technology" OR "Wearable Devices" OR "Biomonitoring" OR "Artificial Intelligence") AND ("Military Applications" OR "Defense Technology" OR "Military Operations") | 117 Studies identified |

**4. Search Filters Applied**

- **Database:** PubMed/MEDLINE (NCBI)
- **Language:** English
- **Publication Date:** 2000-present
- **Document Type:** No restriction applied (all study types included at this stage)
- **Access Type:** No access restrictions were applied. Articles behind paywalls will be accessed via institutional library resources or interlibrary loan services where needed.

**Table A 2. Scientific databases, grey literature, and governmental/military sources used in the search strategy**

| **Category** | **Database / Platform** | **Provider / Organization** | **Access Link** |
| --- | --- | --- | --- |
| **Scientific Databases** | **PubMed/ MEDLINE** | National Center for Biotechnology Information (NCBI) | [pubmed.ncbi.nlm.nih.gov](https://pubmed.ncbi.nlm.nih.gov/) |
|  | **Web of Science** | Clarivate Analytics | [www.webofscience.com](https://www.webofscience.com/) |
|  | **Scopus** | Elsevier | [www.scopus.com](https://www.scopus.com/) |
|  | **IEEE Xplore** | Institute of Electrical and Electronics Engineers (IEEE) | [ieeexplore.ieee.org](https://ieeexplore.ieee.org/) |
| **Grey Literature** | **CyberLeninka** | Independent academic repository | [cyberleninka.ru](https://cyberleninka.ru/) |
|  | **eLIBRARY.ru** | Russian Science Citation Index | [elibrary.ru](https://www.elibrary.ru/) |
| **Governmental / Military Sources** | **NATO** (North Atlantic Treaty Organization) | NATO Science & Technology | [www.nato.int](https://www.nato.int/) |
|  | **WHO** (World Health Organization) | World Health Organization (WHO) | [www.who.int](https://www.who.int/) |
|  | **DARPA** (Defense Advanced Research Projects Agency) | U.S. Department of Defense | [www.darpa.mil](https://www.darpa.mil/) |
